# COVID‐19 Vaccination and Health Outcomes Among Adults With an Intellectual Disability in British Columbia, Canada

**DOI:** 10.1111/jir.70079

**Published:** 2026-01-21

**Authors:** Xibiao Ye, Shengjie Zhang, Marie Paul Nisingizwe, Ioana Sevcenco, Henry Ngo, Alyssa Parker, Yao Nie, Aanu Abayomi, Ross Chilton, Bonnie Henry, Daniele Behn‐Smith

**Affiliations:** ^1^ Office of the Provincial Health Officer, Ministry of Health, lək̓ʷəŋən Territory Victoria British Columbia Canada; ^2^ School of Health Information Science University of Victoria, lək̓ʷəŋən & W̱SÁNEĆ Territories Victoria British Columbia Canada; ^3^ Data Science and Health Research Cluster, Faculty of Medicine University of British Columbia, xʷməθkʷəy̓əm (Musqueam) Territory Vancouver British Columbia Canada; ^4^ Community Living British Columbia, xʷməθkʷəy̓əm (Musqueam), Sḵwx̱wú7mesh (Squamish), and səlilwətaɬ (Tsleil‐Waututh) Territories Vancouver British Columbia Canada; ^5^ School of Population and Public Health University of British Columbia, xʷməθkʷəy̓əm (Musqueam) Vancouver British Columbia Canada

**Keywords:** COVID‐19, intellectual disability, SARS‐CoV‐2 virus, vaccination

## Abstract

**Introduction:**

Early studies demonstrated a higher risk for SARS‐CoV‐2 virus infection and severe COVID‐19 outcomes such as hospitalisation, intensive care unit admission and death among people with an intellectual disability or other chronic conditions. However, the extent to which COVID‐19 vaccination has affected the risk of these outcomes remains unclear.

**Methods:**

We conducted a case–control study to examine the association between vaccination and SARS‐CoV‐2 virus infection risk in people with an intellectual disability and the general population. COVID‐19 cases aged 19 years and older confirmed to be infected between 28 January 2020 and 31 December 2021 were obtained from the British Columbia (bc) COVID‐19 Integrated Case List, and up to five controls were selected from the province's healthcare client registry matching on sex, age and residential region. COVID‐19 vaccination status was determined using the province's immunisation registry. The Community Living bc (CLBC) Registry of supported adults was linked to identify the intellectual disability status of each case and control. Odds ratios (OR) and 95% confidence intervals (95% CI) were estimated using the conditional logistic regression model. Severe COVID‐19 outcomes were ascertained using hospitalisation and death registry databases. Cox regression was used to estimate hazard ratios (HRs) and 95% CIs for the outcomes among COVID‐19 cases with and without an intellectual disability. We adjusted for sex, age, residential area and comorbidities.

**Results:**

CLBC‐supported adults with an intellectual disability were less likely to contract SARS‐CoV‐2 virus (OR = 0.66, 95% CI 0.61–0.71), and the protective association was stronger when fully vaccinated (OR = 0.40, 95% CI 0.34–0.48). Once infected, adults with an intellectual disability had a higher risk for COVID‐19–associated hospitalisation (HR = 1.96, 95% CI 1.60–2.39), ICU admission (HR = 1.61, 95% CI 1.10–2.36) and death (HR = 1.88, 95% CI 1.15–3.07).

**Conclusion:**

COVID‐19 vaccination was effective in reducing the risk of SARS‐CoV‐2 virus infection among people with an intellectual disability. Safety measures such as prioritised vaccination are important steps for protecting vulnerable people with an intellectual disability from SARS‐CoV‐2 virus infection, especially as COVID‐19 cases with an intellectual disability were more likely to suffer severe health outcomes.

## 1 Background

In British Columbia (bc), disability is defined as an inability to participate fully and equally in society as a result of the interaction of an impairment and a barrier (Government of British Columbia 2021). Fewer than 1% of Canadians live with an intellectual disability, with estimates ranging between 0.2% and 0.8% depending on methodology used (Bielska et al. 2012; Lin et al. 2013; Ouellette‐Kuntz et al. 2009). Adults with an intellectual disability are more likely to experience unmet healthcare and social support needs, often as a result of ableism (Lundberg and Chen 2023), which contributes to poorer health outcomes and a lower quality of life (Videlefsky et al. 2019).

During the early stage of the COVID‐19 pandemic, self‐advocates, families, service providers, home‐sharing providers and experts raised concerns that people with disabilities, including intellectual disabilities, are at higher risk of contracting SARS‐CoV‐2 virus and developing severe complications. Early studies verified the concerns, showing that adults with severe intellectual disabilities were at a higher risk of SARS‐CoV‐2 virus infection and of being hospitalised for and dying from COVID‐19–associated complications after contracting the virus (Landes et al. 2020; Turk et al. 2020). The higher risks were confirmed later by studies conducted in the United Kingdom (Baksh et al. 2022, 2021; Sosenko et al. 2023). The evidence demonstrated a clear need for enhanced clinical care and prioritised access to COVID‐19 vaccines for people with an intellectual disability.

Consequently, governments were urged to prioritise COVID‐19 vaccination for people with intellectual disability and vulnerable groups (Epstein et al. 2021; Hotez et al. 2021; Rotenberg et al. 2021). In bc, a COVID‐19 vaccination programme was first rolled out in December 2020 with the first high‐risk group including healthcare workers, long‐term care facility residents and Indigenous people in remote and isolated communities. The subsequent phase prioritised individuals deemed clinical extremely vulnerable, including people with significant developmental and intellectual disabilities, specific forms of cancers, severe respiratory conditions, rare blood diseases, diabetes, history of organ transplants and other immunocompromising conditions. However, little is known about the effectiveness of the vaccination prioritisation for this and other high‐risk groups.

This study aimed to assess the risk of SARS‐CoV‐2 virus infections, COVID‐19–associated hospitalisations and deaths among adults with an intellectual disability in bc compared to the general population without an intellectual disability and to examine the impact of the COVID‐19 vaccination on these risks.

## 2 Methods

### 2.1 Study Population and Design

We conducted a case–control study to examine the associations between COVID‐19 vaccination and SARS‐CoV‐2 virus infection risk among people with and without an intellectual disability based on whether they received CLBC care (Figure S1). Infection cases reported during 28 January 2020 (when the first case was confirmed) and 31 December 2021 were included. For each case, up to five controls were randomly selected from the registered residents aged 19 and older, matching on sex, age at the date of infection (which serves as the index date for controls) and residential region. We used a cohort study design to compare COVID‐19–associated severe outcomes including hospitalisation, intensive care unit (ICU) admission and death between cases with and without an intellectual disability. Hospitalisations with a corresponding COVID‐19–related diagnostic code (U071 or U072) within 30 days of the first COVID‐positive case date were identified. Deaths with the underlying cause of death (UCOD) assigned as ICD‐10 code U071 or U072 within 60 days of the first COVID‐19–positive test date were included.

### 2.2 Data Sources

#### 2.2.1 Community Living bc (CLbc) Registry

CLBC supports BC residents aged 19 and older with an intellectual disability or significant limitation in adaptive functioning (i.e., those who do not have an intellectual disability but have either a diagnosis of foetal alcohol spectrum disorder [FASD] or autism spectrum disorder [ASD] and are cared for under the Personalised Supports Initiative [PSI]). The CLBC registry contains patient demographics, eligibility type (disability diagnosis), service type and residence category. For this study, we excluded patients with FASD or ASD but included 23 671 CLBC‐supported adults with an intellectual disability who were 19 years or older between 28 January 2020 and 31 December 2021.

#### 2.2.2 COVID‐19 Integrated Case List

We used the COVID‐19 Integrated Case List to identify COVID‐19 cases. This database contains all COVID‐19 cases, including laboratory‐confirmed, laboratory‐probable and epidemiology‐linked cases, according to the British Columbia Centre for Disease Control (bcCDC 2021) definitions. For individuals with more than one COVID‐19–positive test, only the first positive test date was considered. All individuals with COVID‐19–negative test results, as well as those who were not tested for COVID‐19 were considered COVID‐19 negative. The database also includes COVID‐19 cases' hospitalisation and death status, which was ascertained through the hospital discharge abstract database (DAD) and the bc Vital Statistics Agency's death registry, respectively.

#### 2.2.3 COVID‐19 Vaccination

COVID‐19 vaccination records were consolidated from three data holdings: PharmaNet (a database of community pharmaceutical dispenses), Provincial Immunisation Registry (PIR, immunisation records from the provincial public health information system Panorama) and ImmsBC (a database of vaccine inventory and immunisation bookings). COVID‐19 vaccination status was determined using COVID‐19 vaccination histories including vaccine name, administration date and dose. Receiving one dose of Janssen vaccine or two doses of vaccines from other manufacturers (provided the second dose was given 14 days or more prior to the date of infection) was considered fully vaccinated. Receiving one dose of non‐Janssen vaccines or the second dose within 14 days of infection was considered partially or not fully vaccinated. SARS‐CoV‐2 virus infection date is defined as the earliest available date among surveillance date (the best date on which the case became known to public health and is populated based on what data are available in each record), laboratory result date (the date when the most recent test result was entered into the laboratory information system), laboratory sample collection date (the date when the specimen was collected) and case symptom‐onset date (the date that the case reports on which the first symptom started). The infection date of a case serves as the index date when determining the corresponding controls' COVID‐19 vaccination status.

#### 2.2.4 Ministry of Health Administrative Datasets

bc has a universal health system like other provinces and territories in Canada. bc residents (citizens or permanent residents living in bc for at least 6 months) must register with the province's public health insurance plan called medical service plan (MSP). The Ministry of Health Client Roster contains information on people registered with MSP. Each bc resident enrolled in MSP is given a unique lifetime identifier called personal health number (PHN). Participants who were not CLbc‐supported adults but included in this study were selected from this data source. We used the BC Chronic Disease Registry (BCCDR) to determine the number of coexisting chronic diseases. BCCDR is a database of individuals with one or more common chronic diseases identified based on healthcare service histories including medical visits (physician visits and hospitalisations) and medication prescription records. More than 99% of individuals in those datasets were linked via their unique PHN.

### 2.3 Statistical Analysis

For the case–control study on SARS‐CoV‐2 virus infection risk, we estimated odds ratios (ORs) and 95% confidence intervals (95% CIs) for intellectual disability using a conditional logistic regression model, adjusting for sex, age group, residential region and the number of comorbidities. To evaluate the association by COVID‐19 vaccination status, we conducted a stratified analysis by the COVID‐19 fully vaccinated status, using cases diagnosed between 1 May 2021 and 31 December 2021. To test the robustness of the analysis, we conducted the following sensitivity analyses: We included fully vaccinated status as an additional covariate in the logistic regression model using cases diagnosed between 1 May and 31 December 2021; we repeated the modelling using cases diagnosed between 1 January 2021 and 31 December 2021, defining immunisation status as completing two doses of vaccination; we extended the cut‐off for hospitalisation and ICU admission to 60 days.

For COVID‐19–associated severe outcomes, we first ran a Kaplan–Meier analysis to examine the differences in survival curves for hospitalisation, ICU admission and death between the groups with and without an intellectual disability (Figure S2). We calculated hazard ratios (HRs) and 95% CIs for each outcome using the proportional hazard model (Cox model), adjusting for sex, age group, residential region and the number of comorbidities (e.g., diabetes, cardiovascular diseases, chronic kidney disease, hypertension, chronic respiratory diseases, chronic neurologic conditions, mental illness and substance uses, and chronic skeletomuscular diseases). All analyses were conducted using R Version 4.2.2.

## 3 Results

There were, in total, 194 005 SARS‐CoV‐2 virus infection cases in the province between January 2020 and December 2021. No statistically significant differences were observed between the case and control groups regarding demographics or other characteristics, with the exception of the number of comorbidities (Table 1). Of the cases, 907 were adults currently receiving CLBC intellectual disability care. Cases with and without an intellectual disability were different in the distributions of sex, age, number of comorbidities and residential area.

**TABLE 1:** Comparisons of characteristics of COVID‐19 cases and controls.

Characteristics	COVID‐19 cases			Controls (*N* = 970 025)
	With intellectual disability (*N* = 907)	Without intellectual disability (*N* = 193 098)	Total (*N* = 194 005)	
Sex (% of males)^a^	503 (55.46%)	95 767 (49.60%)	96 270 (49.62%)	481 350 (49.62%)
Median age (range)^a^	30 (18, 90)	39 (18, 107)	39 (18, 107)	39 (18, 113)
*N* of comorbidities^a^ ^,^ ^b^				
0	133 (14.66%)	72 593 (37.59%)	72 726 (37.49%)	407 621 (42.02%)
1	164 (18.08%)	31 448 (16.29%)	31 612 (16.29%)	153 478 (15.82%)
2	180 (19.85%)	35 794 (18.54%)	35 974 (18.54%)	178 450 (18.40%)
3+	430 (47.41%)	53 263 (27.58%)	53 693 (27.68%)	230 476 (23.76%)
Health authority^a^				
Interior	175 (19.29%)	27 103 (14.04%)	27 278 (14.06%)	136 390 (14.06%)
Fraser	374 (41.23%)	95 680 (49.55%)	96 054 (49.51%)	480 270 (49.51%)
Vancouver coastal	145 (15.99%)	42 322 (21.92%)	42 467 (21.89%)	212 335 (21.89%)
Vancouver island	87 (9.59%)	13 421 (6.95%)	13 508 (6.96%)	67 540 (6.96%)
Northern	126 (13.89%)	14 572 (7.55%)	14 698 (7.58%)	73 490 (7.58%)
With an intellectual disability^b^			907 (0.47%)	6309 (0.65%)
Fully vaccinated for COVID‐19^b^				
Yes			48 222 (24.86%)	302 322 (31.17%)
No			145 783 (75.14%)	667 703 (68.83%)
Number (%) hospitalised^a^	99 (10.92%)	11 951 (6.19%)		
Number (%) admitted to ICU^a^	27 (2.98%)	3668 (1.90%)		
Number (%) of death	16 (1.76%)	3167 (1.64%)		

^a^ Statistically different between cases with and without an intellectual disability, using the Welch two‐sample *t*‐test for age or Pearson's chi‐squared test for other variables.

^b^ Statistically different between cases (total) and controls, using the Welch two‐sample *t*‐test for age or Pearson's chi‐squared test for other variables.

Overall, a lower risk of SARS‐CoV‐2 virus infection was associated with receiving CLBC intellectual disability care (OR = 0.66, 95% CI 0.61–0.71) after adjusting for potential confounders (Table 2). When examined by COVID‐19 vaccination status (Table 2), we observed a smaller OR for SARS‐CoV‐2 virus infection (OR = 0.40, 95% CI 0.34–0.48) among those fully vaccinated, compared to the general population without an intellectual disability; the association remained among those who were not fully vaccinated (OR = 0.74, 95% CI 0.64–0.87). COVID‐19 vaccination was associated with a lower SARS‐CoV‐2 virus infection risk overall (OR = 0.48, 95% CI 0.47–0.49), the association was stronger among those with an intellectual disability (OR = 0.23, 95% CI 0.19–0.29) (Table 3).

**TABLE 2:** Odds ratio (OR)^a^ for SARS‐CoV‐2 virus infection comparing adults with an intellectual disability to the general population.

Vaccination status	With intellectual disability?	Case	Control	OR (95% CI)
Overall^a^, ^c^	Yes	907 (0.5%)	6309 (0.7%)	0.66 (0.61, 0.71)
	No	193 098 (100%)	963 716 (99%)	—
Fully vaccinated^b^, ^c^	Yes	139 (0.3%)	2199 (0.7%)	0.40 (0.34, 0.48)
	No	47 968 (100%)	296 717 (99%)	—
Not fully vaccinated^b^, ^c^	Yes	261 (0.6%)	1078 (0.7%)	0.74 (0.64, 0.87)
	No	44 014 (99%)	161 916 (99%)	—

^a^ Analysis using all COVID‐19 cases confirmed by 31 December 31 2021.

^b^ Analysis using cases confirmed between 1 May 2021 and 31 December 2021.

^c^ Conditional logistic regression adjusted for number of comorbidities (0, 1, 2, 3+), sex, age group and residential region.

**TABLE 3:** ORs^a^ ^,^ ^b^ between COVID‐19 vaccination status and SARS‐CoV‐2 virus infection in adults with an intellectual disability and the general population.

With intellectual disability?	Fully vaccinated	Case	Control	OR (95% CI)
Overall^b^, ^c^	Yes	48 107 (52%)	298 916 (65%)	0.48 (0.47, 0.49)
	No	44 275 (48%)	162 994 (35%)	—
Yes^b^, ^c^	Yes	139 (35%)	2199 (67%)	0.23 (0.19, 0.29)
	No	261 (65%)	1078 (33%)	—
No^b^, ^c^	Yes	47 968 (52%)	296 717 (65%)	0.48 (0.48, 0.49)
	No	44 014 (48%)	161 916 (35%)	—

^a^ Analysis using cases confirmed between 1 May 2021 and 31 December 2021.

^b^ Adjusted for number of comorbidities (0, 1, 2, 3+), sex, age group and residential region.

^c^ Conditional logistic regression.

COVID‐19 cases with an intellectual disability were more likely to be hospitalised and to be admitted to ICU than those without an intellectual disability (Table 4). Of the 907 COVID‐19 cases with an intellectual disability, 99 (10.9%) were hospitalised, compared to only 6.2% of the cases without an intellectual disability. After confounders were adjusted for, the HR for hospitalisation is 1.96 (95% CI 1.60–2.39). Twenty‐seven of the 907 COVID‐19 cases with an intellectual disability (3.0%) were admitted to ICU, a higher proportion than the 1.9% of cases without an intellectual disability, resulting in an adjusted HR of 1.61 (95% CI 1.10–2.36). The risk of death among those with an intellectual disability was also higher, with an adjusted HR of 1.88 (95% CI 1.15–3.07). Cases with an intellectual disability had a higher risk for COVID‐19–associated hospitalisation regardless of their COVID‐19 vaccination status; however, the data were insufficient for a stratified analysis for ICU admission and death.

**TABLE 4:** Hazard ratio^a^ ^,^ ^b^ for COVID‐19–associated hospitalisation, intensive care unit admission and death comparing adults with an intellectual disability to the general population.

Vaccination status	With intellectual disability?	N of COVID‐19 cases (194 005)	Hospitalisation		ICU admission		Death	
			*N* (12 050)	HR (95% CI)	*N* (3695)	HR (95% CI)	*N* (3183)	HR (95% CI)
Overall^a^, ^c^	Yes	907	99	1.96 (1.60, 2.39)	27	1.61 (1.10, 2.36)	16	1.88 (1.15, 3.07)
	No	193 098	11 951	—	3668	—	3167	—
Fully vaccinated^b^, ^c^	Yes	139	8	2.12 (1.06, 4.26)	0	—	0	—
	No	47 968	1209	—	253	—	440	—
Not fully vaccinated^b^, ^c^	Yes	261	33	1.77 (1.26, 2.50)	11	1.88 (1.04, 3.42)	4	1.88 (0.70, 5.04)
	No	44 014	4184	—	1430	—	771	—

^a^ Analysis using all COVID‐19 cases confirmed by 31 December 2021.

^b^ Analysis using cases confirmed between 1 May 2021 and 31 December 2021.

^c^ Adjusted for number of comorbidities (0, 1, 2, 3+), sex, age group and residential region.

## 4 Discussion

Our study found that the population with an intellectual disability reflected in the CLBC registry experienced a lower risk of SARS‐CoV‐2 virus infection both before and after being fully vaccinated, compared to the general population without an intellectual disability during 2020–2021. Landes et al. (2021c) examined the association between intellectual disability and SARS‐CoV‐2 virus infection using the California Department of Developmental Disability Services data linked to COVID‐19 data and found a lower infection rate among those receiving intellectual disability services compared to those not receiving the services, i.e., 0.831% vs. 2.085%. A later study with the same study design but including seven additional states verified the association, showing 5.62% infection rate among residents with an intellectual disability compared to 7.57% among those without an intellectual disability (Davis et al. 2021). However, other studies (Gleason et al. 2021; Landes et al. 2020; Lunsky et al. 2022) have found that people with an intellectual disability were more likely to be infected with the virus, with a COVID‐19 positivity rate ratio ranging from 1.28 (95% CI 1.23–1.34) (Lunsky et al. 2022) to 4.10 (no 95% CI reported) (Landes et al. 2020). The risk for SARS‐CoV‐2 virus infections and COVID‐19 severe outcomes was greater among people with an intellectual disability who were aged 65 or older (Henderson et al. 2022).

These inconsistent findings regarding SARS‐CoV‐2 virus infection risk may be attributed to differences in patient profiles, residential settings and care policies, such as social distancing, isolation and vaccination prioritisation, during the pandemic. In California, SARS‐CoV‐2 virus infection rates varied significantly by residence type (Landes et al. 2021c); those living on their own or in a family home had a lower infection rate than the general population, whereas those living in congregated residence settings, including community care facilities, intermediate care facilities and skilled nursing facilities, had higher rates. Residence type reflects the vulnerability level of patients related to disability complexity and care need level, with those living in skilled nursing facilities requiring 24‐h intensive nursing care. In the present study, 63.3% of people with an intellectual disability lived with a family, while the rest lived with housing support (9.3% with independent living, 16.6% with home sharing and 10.8% with staffed homes), but our sample size is insufficient for a meaningful stratified analysis by residence type.

The present study demonstrates that the COVID‐19 vaccine offered greater protection against SARS‐CoV‐2 virus infection for adults with an intellectual disability than for the general population. Despite the solid evidence on overall efficacy and effectiveness of COVID‐19 vaccines from randomised trials and real‐world studies in the general population (Law et al. 2023; Soheili et al. 2023; Wu et al. 2023), little is known about the efficacy and effectiveness in special populations, as they were often excluded from the studies or their data were not examined separately. The lower risk of SARS‐CoV‐2 virus infection among adults with an intellectual disability who are receiving CLbc care and are fully vaccinated provides evidence on the benefit of vaccination prioritisation for this population in BC.

The estimated numbers of COVID‐19 cases, hospitalisations and deaths averted by COVID‐19 vaccination varied across countries and regions, in some cases very significantly, due to differences in demographics, vaccination coverage and modelling methodology. In Canada, approximately 11 million COVID‐19 cases, 1.1 million hospitalisations and 0.5 million deaths were potentially prevented between December 2020 and March 2022 (Tuite et al. 2023). On a global level, 14.4 million COVID‐19 deaths were prevented in the first year (between December 2020 and December 2021) of vaccine rollout (Watson et al. 2022), but a more conservative approach has resulted in a much lower estimate, i.e., 2.5 million deaths averted during 2020–2024 (Ioannidis et al. 2025). However, most of these estimations were conducted for the general population. Using the odd ratio from this study, we estimated that approximately 20% of COVID‐19 cases in the general population and 50% of the cases among those receiving CLBC services were prevented.

Consistent with studies in Canada and internationally (Bahremand et al. 2023; Cuypers et al. 2023; Lunsky et al. 2022), we found higher risks of severe COVID‐19 outcomes among people with an intellectual disability. Factors such as pre‐existing comorbid conditions, male sex, ethnicity/race and smoking likely contributed to these outcomes (Gao et al. 2021). Earlier studies have found a higher COVID‐19–associated death risk among people with intellectual disability compared to those without. The first study in the United States found comparable overall case–fatality rates in people with and without an intellectual disability, but among those aged 0–17 years old, the case–fatality rate was higher in those with an intellectual disability than in those without (Turk et al. 2020). Other US studies consistently showed a higher case–fatality ratio among those with an intellectual disability (Gleason et al. 2021; Landes et al. 2021c, 2021b, 2020; Davis et al. 2021). This was also confirmed by studies in other jurisdictions, e.g., Ontario, Canada (Lunsky et al. 2022), and the Netherlands (Cuypers et al. 2023; Koks‐Leensen et al. 2023). Previous studies demonstrated that COVID‐19 vaccination offered protection against severe outcomes (Law et al. 2023). In this study, COVID‐19 cases with an intellectual disability were more likely to be hospitalised before and after two‐dose vaccination, similar to the finding in an earlier study in the province where all extremely vulnerable clinical patients (including patients with intellectual disability) were studied (Bahremand et al. 2023).

Our findings highlight the importance of designing response strategies for people with an intellectual disability who are especially vulnerable to pandemics such as COVID‐19. People with disabilities are often disproportionally affected by pandemics due to unequal access to public health messaging, disability service disruptions and additional barriers due to their disability (Armitage and Nellums 2020). While the United Nations and various countries adopted the disability‐inclusive COVID‐19 response strategies to ensure those with disabilities were not left behind and were not experiencing additional inequities (Public Health Agency of Canada 2000; United Nations 2000). There remains a lack of data collection and scientific evidence on the effectiveness of these inclusion strategies (Landes and Turk 2024; Pearce et al. 2022).

In bc, the lower risk of SARS‐CoV‐2 virus infection among CLBC supported adults with an intellectual disability likely reflects the success of targeted measures. These included prioritised access to vaccines, distancing and masking, less congregation in home settings (of home‐sharing providers) and CLBC's proactive communications with self‐advocates, families, service providers and public health officials. Such interventions are critical, as people with disabilities often encounter unique obstacles, including vaccine hesitancy, lack of accessible transport and complex appointment systems (Rattay et al. 2024; Sebring et al. 2022). Ultimately, engaging this population in developing vaccination programmes is essential to enhance accessibility (Sebring et al. 2022). Evidence suggests that strategies incorporating interactive education and direct engagement significantly enhance the acceptability and feasibility of health interventions for adults with an intellectual disability (Bartels et al. 2024).

Our study is a population‐based study using high‐quality linkable health datasets but has limitations. Misclassification cannot be ruled out as we only included those registered for CLBC services. The present study did not look at the impacts among patients with specific intellectual disabilities such as Down syndrome, which some studies suggest is more vulnerable than other intellectual disability patients in the COVID‐19 pandemic (Landes et al. 2021a). Furthermore, the sample size did not permit more stratified analyses, e.g., COVID‐19 infection risk by residential setting, nor did we examine other health impacts, e.g., mental health and post COVID‐19 condition (or long COVID).

In conclusion, the study demonstrates that people with an intellectual disability under the CLBC care had lower odds of SARS‐CoV‐2 virus infection, likely attributed to vaccination prioritisation and provincial safety measures. These findings highlight the importance and the effectiveness of disability‐inclusive pandemic response in reducing the inequities experienced by people with an intellectual disability.

## Funding

The authors received no specific funding for this work.

## Conflicts of Interest

The authors declare no conflicts of interest.

## Supporting information


**Figure S1:** Study design.
**Figure S2:**. Survival curves for hospitalisation (a), ICU admission (b) and death (c) among adults with an intellectual disability and the general population.

## Data Availability

The data that support the findings of this study are available from Ministry of Health. Restrictions apply to the availability of these data, which were used under license for this study. Data are available from the author(s) with the permission of Ministry of Health.
