# Supplementary material for: COVID‐19 Vaccination and Health Outcomes Among Adults With an Intellectual Disability in British Columbia, Canada
Source: J Intellect Disabil Res. 2026 Jan 21;70(4):395–402. doi: 10.1111/jir.70079 (PMC12950626; doi:10.1111/jir.70079)

**COVID-19 Outcomes among Adults with An Intellectual Disability in British Columbia, Canada**

Supplemental Figure 1 Study design

COVID-19 cases

With an intellectual disability?

(CLBC client or not)

Up to 5 controls matched on sex, age, and residential region

With an intellectual disability (receiving CLBC ID care)

(

Without an intellectual disability

COVID-19 associated hospitalisation, ICU admission, and death

**Cohort Study on COVID-19 associated severe outcomes**

**Case-control Study on SARS-CoV-2 virus infection risk**

Supplemental Figure 2, Survival curves for hospitalisation (a), ICU admission (b), and death (c) among adults with an intellectual disability and the general population


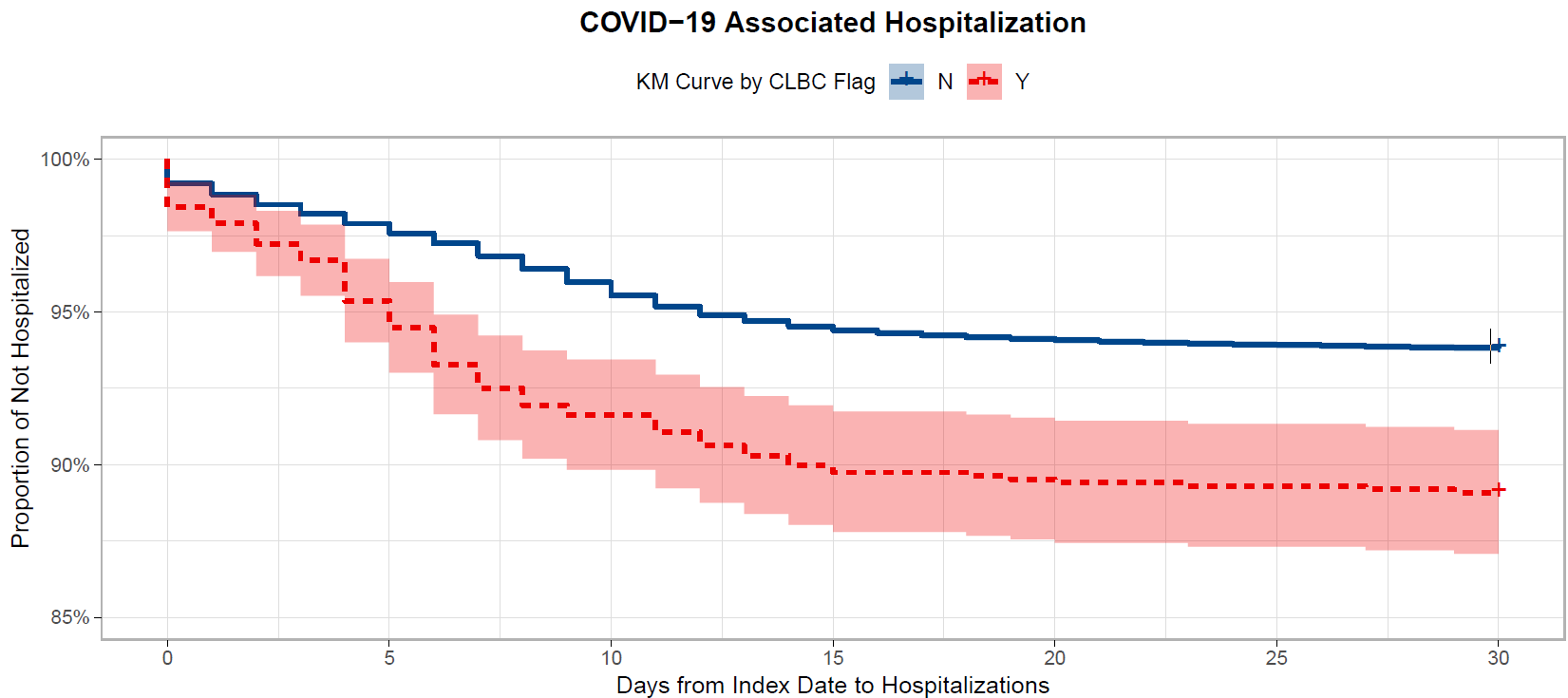


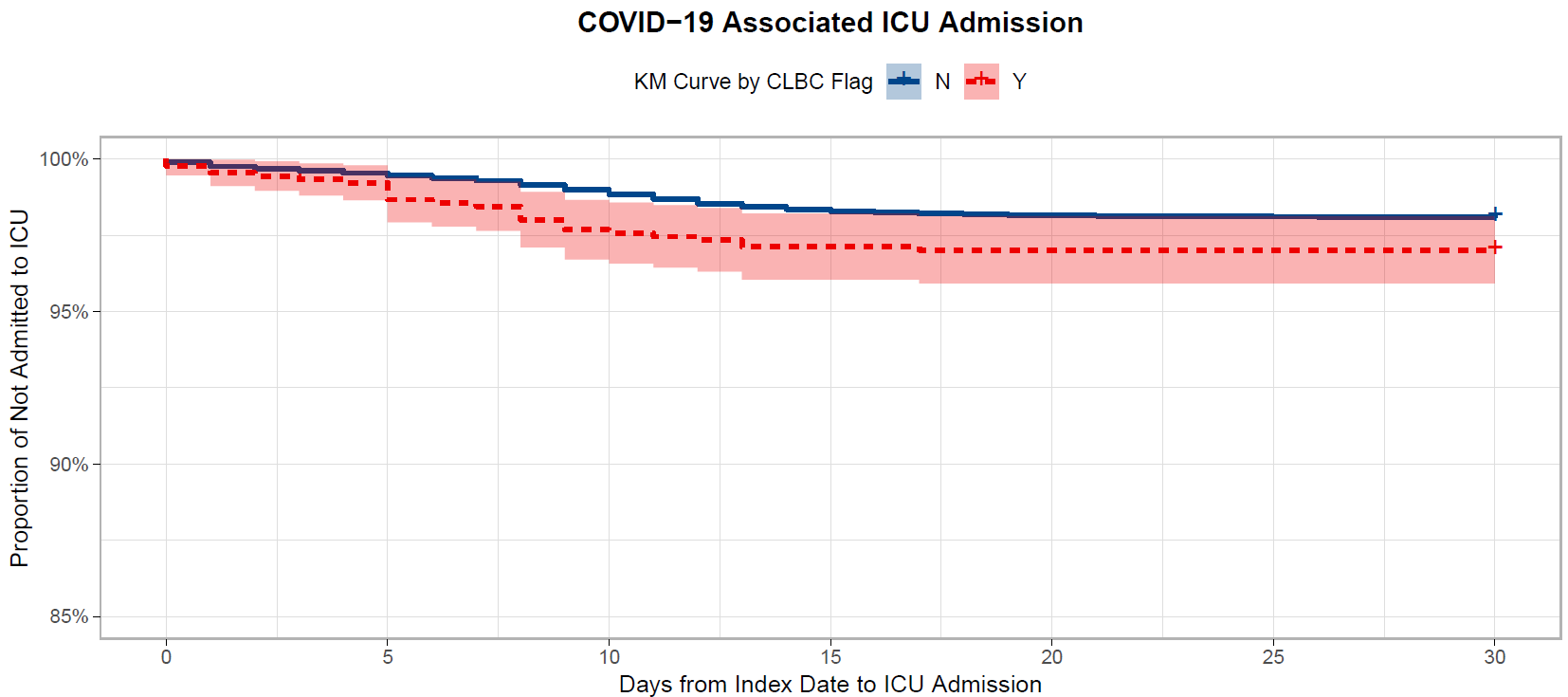


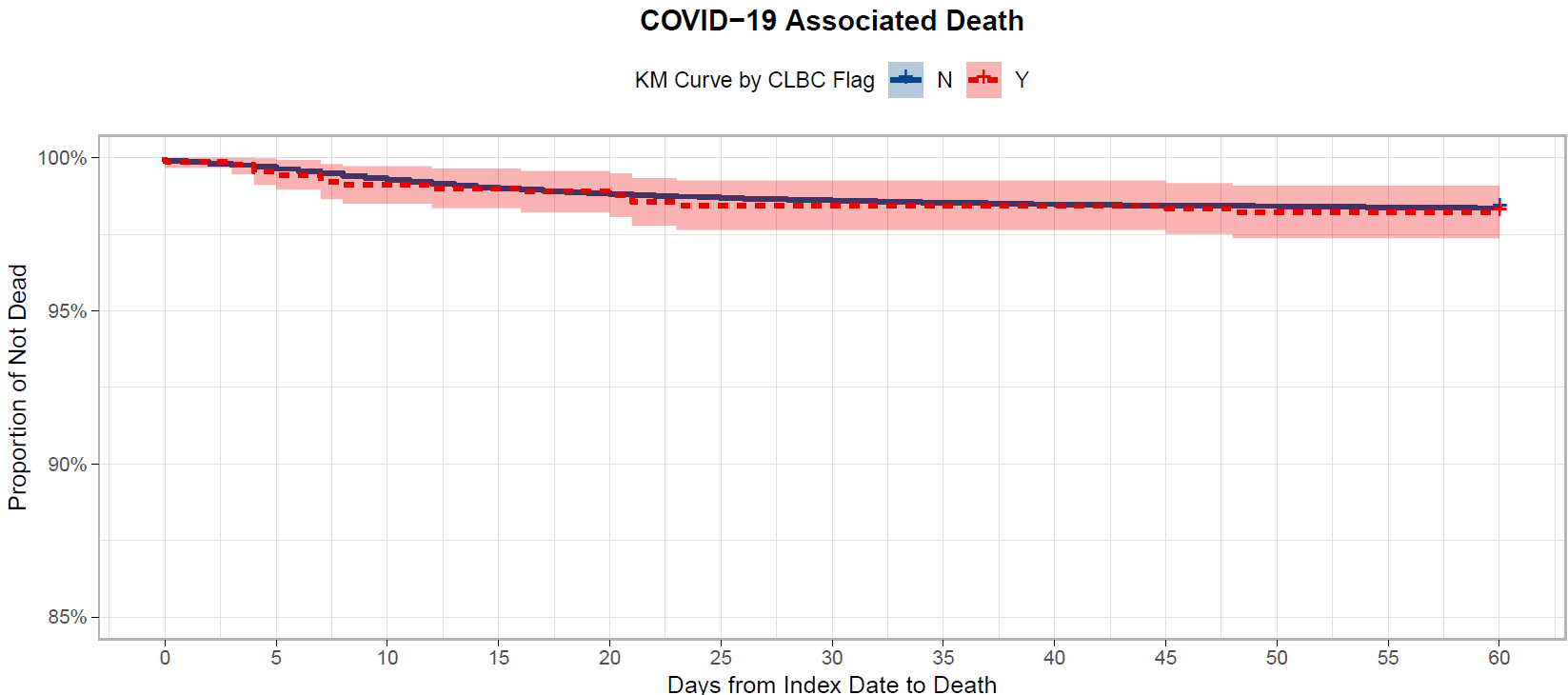

Supplement: Supplementary file 1 — Figure S1: Study design. Figure S2:. Survival curves for hospitalisation (a), ICU admission (b) and death (c) among adults with an intellectual disability and the general population. [file JIR-70-395-s001.docx]
